# Critical care physician cognitive task analysis: an exploratory study

**DOI:** 10.1186/cc7740

**Published:** 2009-03-05

**Authors:** James C Fackler, Charles Watts, Anna Grome, Thomas Miller, Beth Crandall, Peter Pronovost

**Affiliations:** 1 Department of Anesthesiology and Critical Care Medicine, The Johns Hopkins University School of Medicine, 600 North Wolfe Street, Baltimore, MD 21287, USA; 2 Division of Pulmonary and Critical Care, Northwestern University School of Medicine, 251 East Huron, Chicago, IL 60611, USA; 3 Klein Associates, Applied Research Associates, 1750 Commerce Center Blvd, Fairborn, OH 45324, USA

## Abstract

**Introduction:**

For better or worse, the imposition of work-hour limitations on house-staff has imperiled continuity and/or improved decision-making. Regardless, the workflow of every physician team in every academic medical centre has been irrevocably altered. We explored the use of cognitive task analysis (CTA) techniques, most commonly used in other high-stress and time-sensitive environments, to analyse key cognitive activities in critical care medicine. The study objective was to assess the usefulness of CTA as an analytical tool in order that physician cognitive tasks may be understood and redistributed within the work-hour limited medical decision-making teams.

**Methods:**

After approval from each Institutional Review Board, two intensive care units (ICUs) within major university teaching hospitals served as data collection sites for CTA observations and interviews of critical care providers.

**Results:**

Five broad categories of cognitive activities were identified: pattern recognition; uncertainty management; strategic vs. tactical thinking; team coordination and maintenance of common ground; and creation and transfer of meaning through stories.

**Conclusions:**

CTA within the framework of Naturalistic Decision Making is a useful tool to understand the critical care process of decision-making and communication. The separation of strategic and tactical thinking has implications for workflow redesign. Given the global push for work-hour limitations, such workflow redesign is occurring. Further work with CTA techniques will provide important insights toward rational, rather than random, workflow changes.

## Introduction

Physician care provided for hospitalised patients has undergone a dramatic change over the past decade. As one example, the imposition of work-hour limitations on house-staff is believed to be either good [1] or bad [2] and has either imperiled continuity [3] or improved decision-making [4]. Regardless, the structure and function of every physician team in every academic medical centre has been irrevocably altered.

Whether the changes are good or bad is not, however, the appropriate first question. First, there must be an explicit and complete delineation of the goals of the physician team and the necessary requisite tasks performed to meet those goals. For example, the conceptual goal of a critical care unit-based physician team is to bring 16 patients back to their baseline health as quickly and as safely as is possible. Obviously, specific operational goals (e.g. endotracheal extubation, full calorie delivery) must be set. Tasks this team must perform include cognitive tasks (e.g. triaging admissions and deciding whether a white cell count of 24,000 × 10^9^/L with a 38.4°C temperature warrants antibiotics). Tasks also include procedural tasks such as endotracheal intubation and central line placement. A subset of procedural tasks is administrative (e.g. prescribing orders, documentation, scheduling imaging studies).

Sporadic efforts have been made to redistribute some physician tasks. For example, many academic medical centres have created teams to place intravenous catheters. Yet, a comprehensive task analysis has not been performed for physician teams.

The purpose of this study was to determine whether the techniques of cognitive task analysis (CTA) (see Table 1 for definition) guided by the theoretical framework of naturalistic decision-making (NDM) (Table 1) can be used to begin the comprehensive physician-team task analysis to guide physician-team restructuring and/or task reallocation.

**Table 1:** Definitions of terms used in the study.

**Term**	**Definition**
Cognitive Task Analysis	A family of methods for gaining access to the cognitive processes that underlie performance of tasks, and the cognitive skills needed to respond adeptly to complex situations.
Naturalistic Decision Making (paradigm)	The scientific study of decision-making in complex, natural environments.
Dyad	Aa two-person team.
Ethnography	Use of qualitative research methods to provide a detailed, in-depth description of everyday life and practice. The ethnographic approach has its roots in cultural anthropology.

These definitions are crucial to understanding the methods and findings.

## Materials and methods

### Participants

After approval from each Institutional Review Board, two intensive care units (ICUs) within major university teaching hospitals served as data collection sites. Consent was waived given the work used interview procedures and observation of public behavior and no data were personally identifiable. One of the ICUs is a 20-bed medical ICU. The medical team is typically a critical care attending, a fellow, nurse practitioners and rotating internal medicine residents. The second ICU is a 14-bed unit that generally cares for surgical oncology patients. It is staffed by a critical care attending, a fellow, nurse practitioners and rotating anaesthesia and surgical residents. Both teams are supported by a clinical pharmacist. Neither ICU has in-house attending coverage 24 hours a day seven days a week, although the second ICU has 24-hour in-house fellow coverage.

Between the two hospitals, we interviewed 14 members of these medical teams and six bedside nurses who were either rostered to provide clinical care at the time of the study or were physically in the ICU for another reason. The participants included: seven attending physicians, three fellows, two residents, one clinical pharmacist and one nurse practitioner. Observational data were collected over two days in each unit, beginning with morning rounds. The observers were afforded extensive access to the units and their staffs, and all health care providers on the ICU.

### Data collection

A four-person research team carried out the CTA [5] interviews and conducted the ICU observations on two consecutive days at each site. No research team member had special medical training. For this initial work, data collection was focused on three topic areas (chosen by consensus of the authors): cognitive processes and decision-making; technology use; and team issues. Investigation of cognitive processes included exploration of decision-making, uncertainty management, attention management, sensemaking, problem detection, leverage points, planning/replanning and use of mental simulation. Questions about technology centred on how ICU personnel used available information technologies. Questions regarding the health care team focused on roles and functions, information management and communication of common ground (information sharing).

### Observations

Researchers spent 70 man-hours observing rounds and interactions among the ICU staff. In one hospital, morning rounds were conducted in parallel by two attending physicians working with two separate teams; pairs of researchers observed each team. The other hospital used one ICU rounding team. On day one, all four members of the research team observed rounds. On day two, observations were conducted by two researchers, while the other researchers conducted interviews. Depending on patient complexity, rounds lasted between 10 and 60 minutes per patient. Research observers were intentionally unobtrusive and shadowed the rounding team standing on the edge of the group. Research observers made notes but did not attempt to interact with unit staff during this portion of data collection.

Given the exploratory nature of the project, the observational data gathering was not highly structured. The research team took an ethnographic approach (Table 1), observing and taking notes on the verbal exchanges, interactions and information exchanged across the rounding team. Particular attention was paid to information flow and the variety of patient data sources (e.g. supporting technologies, patient charts, handwritten notes, reference guides and forms, diagnostic data) accessed by the team and referred to during case presentation. Notes were taken of the questions posed, responses given, requests for data and by whom. The result was a richly detailed record of the rounding team's interactions, information searches, and problem solving and diagnostic processes.

Immediately following our observations, the research team met briefly to identify events that occurred during rounds for follow-up in subsequent interviews. This allowed better linkage between observational and interview data.

Interviews were conducted with 14 ICU staff for between 1.5 and 2 hours; most were physicians as physician decision-making was the study focus. Interviews were conducted using an adaptation of the Critical Decision Method (CDM) of knowledge elicitation [6,7]. The CDM is a CTA technique that relies on recollection of a specific incident as its starting point. It employs a semi-structured interview format with specific, focused probes to elicit particular types of information from the interviewee. For the purposes of this study, the CDM interview was adapted to allow in-depth exploration of events and slices of incidents observed on the unit during rounds [8].

Interviewers made a rough sketch of the rounding team, including team members, their position within the group and any information technologies we had observed in use. The interviewee was asked to describe the roles and functions of each team member, and to identify what information they get from each person and what information they give to each team member.

Next, a decision, assessment or other event was identified for the interviewee, in which he/she had been an active participant. The interviewee was asked to recount the event from their own perspective and a series of cognitive queries, or probes, were posed about the event. Probes were aimed at aspects of uncertainty management, attention management, sensemaking, problem detection, leverage points, planning/replanning and mental simulation.

In addition to the 14 in-depth interviews, three abbreviated interviews were conducted on the unit with on-duty ICU staff. These individuals were selected because of their availability at the time, although the amount of time they had available was limited. They provided important insights into a number of topics such as shift hand-overs, technology, information flow and physician-nurse communication. Finally, additional in-depth interviews were conducted with three intensivists affiliated with different ICUs in the two hospitals. These interviews provided insights into issues such as how training is currently carried out and how senior physicians use technology during patient care.

### Data analysis

Notes from the 14 in-depth interviews were expanded into text files, and checked for accuracy against the original data records of each interviewer.

A preliminary review was made through the full set of observational notes and interview data to identify a comprehensive set of variables and content categories for inclusion in a more systematic examination. A thematic analysis was then conducted to identify major themes and descriptive categories. The analysis yielded 16 thematic topics. Using category formulation and sorting techniques for data reduction and structuring of the thematic data [9], a smaller set of key categories was identified; these comprise the core findings of the study.

## Results

Five key categories of cognitive activities were identified in the data analysis.

### Pattern recognition

Pattern recognition is a key aspect of critical care expertise and a principal area in which less-experienced physicians need further skill development. Members of the ICU team were observed frequently using the term 'pattern', so patterns is a well-recognised construct. However, when asked to describe what was meant by patterns and to give examples, no clear, consistent definition emerged.

We observed pattern recognition in two forms. One pattern was of a complete 'template' or mental model [10,11]. Asthma is one such complete template based on a minimal history, appearance and breath sounds. A typical asthma mental model includes the constellation of cues of a patient who is in an upright position, sweaty, speaking in one word answers, exhibiting labored breathing and attentive to his or her own breathing. However, such 'classic' complete mental models are uncommon.

The second but distinct cognitive task is the real-time merging of pattern fragments (also called 'packets') into unique (patient specific) mental models. Observed more frequently than identifying the complete template, these packets are recognised as cues that are postulated to be related. It is only through a flexible and dynamic integration of these packets that a complete (or partial) mental model can be created. These mental models are highly context specific. The cue of blood pressure of 80/40 mmHg is quite different in a patient with respiratory failure than in a patient with congestive heart failure, chronic hypertension and high serum troponins.

### Strategic versus tactical thinking

Both strategic thinking (long term, often multi-patient, goal oriented) and tactical thinking (short term, single patient, detail and task oriented) were observed. Particularly in the minds of junior housestaff, strategic thinking was not routine. The focus of their activities tended to be much more tactical as they were immersed in the details of testing and treating the patient and coordinating with members of the staff to get a specific treatment plan delivered. Junior housestaff were occasionally observed struggling when required by the attending physician to transition from tactical to strategic-level thinking. For example, an attending physician expressed displeasure with an intern when learning a tracheostomy the patient was supposed to receive had been postponed by several days. The attending physician was thinking at the strategic level (toward the goal of ICU discharge), while the intern was thinking tactically (substituting an imaging study as it was also on his task list).

### Uncertainty management

Substantial evidence documented uncertainty (from various sources) as a defining feature of critical care medicine. Members of the critical care team may be uncertain, for example, about a patient's missing or erroneous laboratory values. They may be uncertain if a patient's symptoms do not fit a complete pattern or about the underlying cause of a patient's illness. One ICU team was grappling with uncertainty surrounding the declining cognitive functioning of a patient. They asked questions such as, "Why is she continuing to experience decreased cognitive functioning? Is she still sedated, and if so, why are the drugs still in her system? Are there other areas of infection we are missing? What haven't we tested for?" Uncertainty permeates all aspects of critical care medicine.

### Creation and transfer of meaning through stories

A critical cognitive task within strategic thinking appears to be the creation and use of stories. The term 'story' was used explicitly during rounds as an attending asked the intern or resident, "what's the patient's story?". Reference was also made to the patient's 'picture'. Despite differences in terminology, the observational and interview data suggest a common cognitive activity. In both settings, health care teams were developing a framework of causal connections and a central theme that tied the various packets of patient data (medical history, test results, etc.) together in a meaningful way. These stories appear to provide the critical care team with an organising mechanism to make decisions about patient care and treatment. Story creation served to create a mental model as a diagnostic tool, to generate expectations and predictions about a patient's trajectory of illness and a way to catch inconsistencies.

There appear to be two key components of stories in the ICU. Firstly, there is story building: activities involved in constructing meaning and creating the story as a means of understanding the patient (i.e. the coalescing of partial templates into a mental model). Secondly, there is story telling: activities involved in sharing the story as a means of communicating and maintaining a common mental model within the health care team.

These two processes appear to occur in real time, sometimes in an emergent fashion. The mental model, or story, of the patient is created and communicated in the moment, during rounds where different members of the ICU team contribute unique template fragments.

The observations showed story creation and story sharing are skills. As with any skill, it is developed through experience. During rounds, interns and residents often presented a lengthy list of problems the patient was facing and data they had at that point, but seemed to have little sense of how to tie the information together into a coherent mental model.

An additional finding that emerged from the interviews is that the complete story is not captured fully in any one place and, often, not by any one caregiver; rather it emerged over time. Important elements of the story may be missing altogether from the patient record or may be fragmented so that one portion is captured in nursing notes, another in the patients' electronic chart and another in a paper-based consultant's note. Moreover, the overall picture, and its supportive data, may not be adequately communicated during shift hand-overs or between members of the health care team. There was evidence suggesting that shift hand-overs often involve a series of checklists, and it is questionable whether or not the essence of the story is captured in these exchanges. When asked about the consequences of losing parts of the picture/story, physicians and nurses readily acknowledged there are negative consequences for decision-making and quality of patient care.

### Team coordination

It was clear in the observations that the two ICU environments studied were characterized by collaboration, where cognitive work and expertise are distributed among members of a multi-disciplinary team that includes nurses, physicians, respiratory therapists, pharmacists, and other professionals.

### Team communication

During the interviews, one of the themes that emerged was the presence of communication difficulties between nurses and physicians. Communication problems between nurses and physicians are complicated, in part, because physicians and nurses believe they speak different languages. Nurses interviewed explained that nurses and physicians use different terminology to refer to patient needs. Their distinct professional backgrounds and training lead to different ways of seeing the world, different concerns and different goals.

### Fragmentary teams

Another complicating factor observed that can impede a common understanding (often called common ground) in the ICU team relates to the sub-teams or smaller dyads (Table 1) that communicate and make important assessments and decisions about patient care throughout the course of the day. Fragments of the larger ICU team – the nurse with the intern, the intern with the pharmacist, etc – were observed engaging in important conversations, during and outside of rounds. In some of these cases, notes from the conversations were recorded. In others, they were not. In some cases these smaller teams were discussing specific tests and specific pieces of data, but were not aggregating it with other data to see the bigger picture. Fractured teams were observed during rounds when team members were interrupted by phone calls, visiting consultants or other ICU issues.

### Shifting teams

Challenges in coordinating and maintaining common ground in the ICU were also found to stem from the unstable and shifting nature of the teams. ICU teams perceive their performance to be negatively impacted by the fact that they change every two to four weeks as residents and attendings rotate in and out.

### Increasing numbers of shift hand-overs

Clinicians interviewed indicated that in many shift hand-overs, important information is not communicated. Critical aspects of the patient picture or story can slip through the cracks, hindering the team's ability to make sense of the patient's condition and to make vital decisions about treatment and care.

### Role ambiguity in the ICU

Confusion surrounding roles and functions in the critical care team were uncovered. These confusions were particularly pronounced surrounding where a nurse's role ends and a physician's role begins. A relatively common sentiment among the nurses was captured in one nurse interview. She explained that sometimes she is asked to use her own discretion regarding certain decisions such as sedation. But when she does use her discretion, the physicians then tell her not to give the patient any more sedatives. As a result, she is frequently unsure about what to do and she questions whether certain decisions are truly at her discretion.

### External collaborators in critical care

During the data collection, difficulties were observed resulting from coordination problems between members of the ICU team and the consultants. It was clear from the observations in both ICUs that the critical care team plays a pivotal role within the context of the larger organisation. A host of challenges arise when members of the ICU team must coordinate patient care and maintain common ground with other members of the larger multi-disciplinary team.

## Discussion

In this exploratory study, CTA tools were applied to identify cognitive aspects of critical care practice in two academic ICUs. CTA is a tool within the theoretical perspective of NDM. Several cognitive activities that members of the critical care team engage in, as part of their decision-making process, were identified including pattern recognition, uncertainty management, strategic vs. tactical thinking, team coordination, creation and transfer of meaning through stories, and maintenance of common ground.

Researchers using the NDM framework have examined expert performance with a wide variety of professionals such as firefighters [12], weapons directors [13], anti-air warfare command and control officers [14], pilots [15], electronic warfare officers [16] and critical care nurses [17]. By studying the cognitive aspects of expert performance in these domains, NDM researchers have been able to make recommendations on how to improve training and system support to facilitate performance of both experts and non-experts.

CTA comprises a series of techniques for knowledge elicitation and knowledge representation ranging from the CDM [7,8,17,18] to the Knowledge Audit [19] to a variety of team CTA techniques [20]. The tools allow an understanding of the cognitive aspects of the expert's behaviour; in particular the judgment, decision-making and problem-solving skills that are so critical in the time-pressured, uncertain and ever-changing environment of the ICU.

These data are neither the first use of cognitive psychology techniques nor cognitive task analysis in particular, within the domain of critical care. Cohen and colleagues used the theoretical framework of distributed cognition (also used extensively in aviation and the military) to examine cognitive errors in a busy psychiatric emergency department [21]. They discovered a number of worrisome system problems associated with cognitive tasks distributed across people, time, space and technologies. One clear similarity between their findings and our own is the concept of fragmentary (or 'mini') teams as sources of cognitive dissidence and potential errors [21]. Others have used observations and interviews to model emergency room hand-over [22] and critical care workflow [23]. All three studies focused on creating systems and technologies to support current workflows. Again, working to support existing workflow, Ho and colleagues observed communication and sense-making during critical care rounds [24]. Closest to the intent of our work is a study by Renard and colleagues where the tasks associated with medication prescribing were analysed, simulated and postulated amenable to redesign [25].

There are several key cognitive areas where this exploratory work suggests deeper analysis will be fruitful and necessary.

### Assistance with mental model creation

Pattern recognition is challenging because of the massive quantities of data with which critical care practitioners are faced. Within a sea of data, it can be extremely challenging to pick up a 'signature' or a pattern. For pattern recognition to be enhanced, better delineation of templates within critical care need to be identified with their essential data elements, and these templates must be explicitly taught to trainees. Rather counterintuitively, the actual number of data elements may be rather small [26,27]. Only after consistent patterns are discovered can their use by the physicians and nurses be studied.

A robust notion of mental models, that takes into consideration causal connections and the meaning of those connections in a given context, is a concept called fragmentary mental models [28]. Fragmentary mental models are packets of local cause/effect connections that permit people to connect smaller pieces into a bigger picture. Fragmentary mental models are put together as the situation warrants to construct a comprehensive just-in-time mental model of the situation – one that is flexible, dynamic (changing over time) and context-specific [28,29]. Experts have a richer and broader repertoire of fragmentary mental models than novices, helping them to draw inferences and to make sense of situations.

### Supporting shifts between, or separation of, strategic and tactical thinking

Both tactical and strategic thinking are central to critical care medicine. Strategic thinking enables the practitioner to consider the big picture (single patient, multi-patient and/or multi-unit), recognise patterns and trends, set priorities and consider alternatives. In a traditional academic setting strategic thinking is generally the responsibility of the attending position and tactical thinking (e.g. running the tests, assessing laboratory values and coordinating with other members of the team) is the responsibility of the housestaff. Being able to transition between tactical and strategic thinking is central to effective critical care medicine as it is currently practiced; yet this is no simple task. Most of the training of residents is focused on tactical thinking. As a result of not operating at the strategic level, it can be difficult for housestaff to effectively prioritise tasks.

### Supporting uncertainty management

It is often assumed that uncertainty stems from a lack of information. If that were the case, the problem of uncertainty could be solved by technological advancements that provide practitioners with access to all possible data. Yet, uncertainty is, in fact, a lot more complex than just missing information [30]. It is often the complexity of the data and the difficulty in integrating the data that leads to uncertainty.

Although physicians may feel more confident in their decision-making when they have more information available, evidence suggests that decision-making performance actually declines with too much data [26,30,31]. More data can simply confuse the issues, making it difficult for physicians to integrate and/or interpret it [26,31]. In addition, the overwhelming majority of information is typically irrelevant to the immediate problem and can get in the way of effective decision-making.

There are practical ramifications of uncertainty in the context of the discussion of templates, patterns and mental models. Appreciating uncertainty is crucial to avoid being locked into an incomplete and, possibly, incorrect, mental model (a cognitive error sometimes called 'premature closure').

### Support creation and transfer of meaning through stories

Related to common ground is the use of stories in the ICU. In many circumstances, important information is being lost in the transition of care from shift to shift. Recent data suggests that because housestaff work restricted work hours, shift hand-over errors exceed the number of errors due to fatigue [32]. This finding suggests critical elements of the patient's story are not being communicated. A broader understanding of stories in the ICU – including the function they serve, how they are created and shared within the team, and how technology can support those processes – may offer significant leverage toward ameliorating this problem.

### Assisting team coordination and common ground

Over the past 10 years, the health care domain has adopted lessons that were learned from the aviation industry, incorporating crew resource management principles to address the challenges of teamwork. A different way to think about the cognitive challenges of teamwork is through the concept of common ground [33]. In this exploratory study, some of the barriers to common ground were identified that ICU teams face such as role ambiguity, the shifting nature of the teams and a higher number of shift hand-overs due to hour restrictions.

Redesigning shift hand-overs or rounds procedures may be a way for ICU teams to enhance common ground [34-36]. A central key to sustaining common ground in teams is to catch discrepancies or possible erosions in common ground, and to take preemptive action to avert a potentially disastrous breakdown [37,38]. Modified shift hand-over or rounding procedures should elicit potential discrepancies before they become entrenched. The procedures and technological support systems will most effectively support common ground if they involve the following activities: first, including various clarifications and reminders that can be used as a means of simply validating an assumption or giving team members a chance to challenge assumptions; second, updating others about changes that occurred outside their view or when they were otherwise engaged; third, monitoring the other team members to gauge whether common ground is being seriously compromised and is breaking down, along with actively encouraging all clinical team members to contribute their knowledge; fourth, detecting anomalies that signal a potential loss of common ground; and fifth, repairing the loss of common ground.

The study has several limitations. The study was, by design, exploratory; more questions were raised than answers delivered. Caregiver sample sizes are small and represent only one model of critical care delivery (i.e. the academic medical centre); the findings are not likely to be directly extensible to other practice models. Few units operate with more providers on rounds. Further, there was no systematic effort in this study to ensure a full cross-section of rounding participants. In units run without housestaff the division of strategic and tactical think is not relevant for separation of the cognitive planes, and rather the focus will need to be more effective transitions between them.

This exploratory work indicates that there is much work to be conducted. One pressing issue is to ensure that the primary cognitive issues are identified so that these cognitive tasks are appropriated distributed and then adequately supported (but not only technological support [39]). Another critical concern is with technologies that hinder, rather than help practitioners, due to their complexity and/or poor design. Unfortunately, human error is all too often simply transformed, or even amplified, by technological change, and new demands are levied which afford new ways to make mistakes. Thoughtful, well-tested systems that effectively support clinical teamwork, based on an understanding of the cognitive and environmental demands of clinical teams in the ICU, is the crucial first step toward future technology acceptance.

## Conclusions

We believe we have shown that the techniques of CTA within the framework of NDM is a useful tool to understand the critical care process of decision-making and communication. Five broad categories of cognitive activities were seen in this analysis: pattern recognition, uncertainty management, strategic vs. tactical thinking, team coordination and maintenance of common ground, and creation and transfer of meaning through stories. Deeper analysis of each activity is necessary to properly support (e.g. with technology) and to redesign both cognitive and physical workflows. Before offering solutions, further investigation is crucial into each of these activities and to the implications for team cognition and communication. The lack of understanding and imprecision of cognitive activities is likely to contribute to significant preventable harm.

## Key messages

• CTA is a useful technique to study physician communication and decision-making.

• Five broad categories of cognitive activities were discovered: pattern recognition, uncertainty management, strategic vs. tactical thinking, team coordination and creation of meaning through stories.

• Junior housestaff were observed to be struggling when required to transition from the tactical level (i.e. task oriented) to strategic level (e.g. about ICU bed availability) of thinking.

• There appear to be two key components of stories in the ICU: story building (i.e. creating a story as a means for understanding a patient; and story telling (i.e. using the story as a means of communication).

• We speculate that the lack of understanding and imprecision of cognitive activities is likely to contribute to significant preventable harm.

## Abbreviations

CDM: Critical Decision Method; CTA: Cognitive Task Analysis; ICU: intensive care unit; NDM: Naturalistic Decision Making.

## Competing interests

This work was funded by the Cerner Corporation, Kansas City, MO, USA. At the time the work was performed, JCF worked full-time for Cerner. As there are no commercial products or services discussed in this work, there are no competing interests. TM, AG and BC were, and remain, employees of the Klein Associates Division within Applied Research Associates Inc. They were paid consultants. The remaining authors declare that they too have no competing interests.

## Authors' contributions

All authors assisted in the study design. Further, AG, TM and BC performed the data collection and analysis. PP and CW coordinated the study sites. JCF and CW prepared the final manuscript. All authors approved the final manuscript.
